# Substance P Concentration in Gestational Diabetes and Excessive Gestational Weight Gain and Its Impact on Neonatal Anthropometry

**DOI:** 10.3390/ijms25073759

**Published:** 2024-03-28

**Authors:** Magdalena Niebrzydowska-Tatus, Aleksandra Pełech, Katarzyna Bień, Anna K. Rekowska, Aleksandra Domańska, Żaneta Kimber-Trojnar, Bożena Leszczyńska-Gorzelak, Marcin Trojnar

**Affiliations:** 1 Chair and Department of Obstetrics and Perinatology, Medical University of Lublin, 20-090 Lublin, Poland; apilszyk@gmail.com (A.P.); bozena.leszczynska-gorzelak@umlub.pl (B.L.-G.); 2 Student’s Scientific Association at the Chair and Department of Obstetrics and Perinatology, Medical University of Lublin, 20-090 Lublin, Poland; kaasiabien@gmail.com (K.B.); arekowska@icloud.com (A.K.R.); aleksandradomanska0206@gmail.com (A.D.); 3 Chair and Department of Internal Diseases, Medical University of Lublin, 20-059 Lublin, Poland; marcin.trojnar@umlub.pl

**Keywords:** substance P, gestational diabetes mellitus, excessive gestational weight gain, fetal programming

## Abstract

Fetal programming is a process initiated by intrauterine conditions, leaving a lasting impact on the offspring’s health, whether they manifest immediately or later in life. It is believed that children born to mothers with gestational diabetes mellitus (GDM) and excessive gestational weight gain (EGWG) may be at an increased risk of developing type 2 diabetes mellitus (T2DM) and obesity later in their adult lives. Substance P is a neurotransmitter associated with obesity development and impairment of insulin signaling. Dysregulation of substance P could lead to several pregnancy pathologies, such as preeclampsia and preterm birth. Our study aimed to compare substance P concentrations in serum and umbilical cord blood in patients with GDM, EGWG, and healthy women with a family history of gestational weight gain. Substance P levels in umbilical cord blood were significantly higher in the GDM group compared to the EGWG and control groups. Substance P levels in serum and umbilical cord blood were positively correlated in all groups and the GDM group. A very interesting direction for future research is the relationship between the concentration of substance P in newborns of diabetic mothers and the occurrence of respiratory distress syndrome as a complication of impaired surfactant synthesis. To our knowledge, it is the first study assessing substance P concentration in GDM and EGWG patients.

## 1. Introduction

Fetal programming is a process that occurs prenatally in which intrauterine conditions cause long-term health consequences for the offspring [1,2]. Nutritional, hormonal, and metabolic environments permanently influence and “program” metabolism, physiology, cellular responses, gene expression, or organ function. These factors, however, can be further modified throughout the fetal development period and differ in dynamically growing fetuses from neonates. Programming can have an immediate effect or manifest itself later in life. The molecular background of the programming includes alterations in the epigenome, cell proliferation, and differentiation, but also gene expression, structure, and function [3,4]. It has already been proven that fetal programming influences, among other things, brain development [5], risk of neuropsychiatric disorders [6], glucose-insulin metabolism [7], and cardiovascular pathologies [8]. A hypothesis proposed by Barker in 1990 shows a link between early-life adverse nutrition and increased risk of metabolic disorders, including metabolic syndrome, obesity, diabetes, or insulin insensitivity, as well as the complications associated with them later in life [2]. Several studies reported associations between low birth weight and features of metabolic syndrome. It was suggested that despite malnutrition, a fetus needs to develop essential organs and, therefore, adjust their organism to occurring circumstances through metabolic adaptation. In addition, low birth weight correlates with impaired glucose tolerance. Among obese adults, those born with low birth weight had the most impaired glucose tolerance [7]. Ross et al. mention fetal exposure to endocrine disruptors and maternal over- or undernutrition, obesity, and high-fat diet as an obesity programming factor. Moreover, they report that over- and undernutrition programs increased adipogenesis [3]. Children of mothers with gestational diabetes mellitus (GDM) are at higher risk of occurrence of type 2 diabetes mellitus (T2DM) and cardio-metabolic diseases in the future [9]. Another study reports that babies with low birth weight are six times more likely to develop T2DM [7]. On the other hand, studies also suggest that obesity in pregnancy and excessive gestational weight gain (EGWG) result in an increased risk of obesity and diabetes in the offspring [3]. Hjort et al. reported that epigenome-wide methylation could stand behind the development of metabolic disease in children of women with GDM and obesity [9]. Moreover, programmed appetite dysregulation, linked to obesity in children, is affected by intrauterine growth restriction (IUGR) and maternal high-fat diet as well [3]. A study by Antoniou et al. proved that numerous maternal anthropometric, metabolic, and fetal parameters directly influence fetal anthropometry. Birth weight, weight z-score, BMI, and/or large for gestational age status were positively associated with cord blood high-density lipoprotein (HDL) and glycated hemoglobin (HbA1c) at the first GDM visit and negatively correlated with maternal quantitative insulin sensitivity check index (QUICKI) and HDL at the first GDM visit [10]. Another study shows that maternal hyperglycemia and obesity increase early-life body fat percentage in their offspring. Additionally, in women with GDM, glycemic levels are more influential than their weight itself [11]. Research on this topic is crucial because almost 50% of pregnant women have been overweight or obese already at the start of pregnancy, and GDM is nowadays diagnosed in up to 15–30% of pregnancies [12,13].

Studies list numerous molecules involved in the process of fetal programming. Jennewein et al. underline the impact of maternal disorders, with an emphasis on infections and inflammatory signals on their offspring’s immune disorders and long-term health consequences. Other reports of microbial antigens transferred across the placenta by maternal antibodies suggest their potential role in the development of infant immunity later in life. [14]. Embryotoxic cytokines, such as tumor necrosis factor (TNF), TNF-related apoptosis-inducing ligand (TRAIL), and interferon-gamma (IFN-γ), could participate in the programming of fetal growth restriction or development of metabolic disorders [15]. In GDM, placental deoxyribonucleic acid (DNA) methylation of leptin and adiponectin is decreased on the fetal side of the placenta during hyperglycemia. Houde et al. suggest that this phenomenon reflects the presence of abnormal adiponectin and leptin methylation in other fetal tissues involved in metabolic processes [16]. Increased nutrient supply and fetal hypertrophy, or adiposity likelihood in infancy and adulthood are influenced by high levels of insulin and leptin, as well as insulin-like growth factor 1 (IGF-1) and low levels of adiponectin in obese pregnant women, which stimulate placental mechanistic Target of Rapamycin (mTOR) signaling and promote protein synthesis and mitochondrial function [17]. Studies on murine models revealed that prenatal hyper-androgenization programs disruptions in fatty acid (FA) metabolism and liver function [18]. Moreover, prenatal androgen excess contributes to polycystic ovary syndrome (PCOS), insulin resistance, and dyslipidemia development [18]. Results of the Hokkaido study suggest that increased cord blood adipokines levels could be associated with hyperactivity or inattention [19]. Also, excessive reactive oxygen species (ROS) formed in prenatal events might affect epigenesis and gestational programming [20], whereas maternal vitamin D deficiency increases the risk of “small for gestational age” newborns [21]. Impairment of adenosine kinase in GDM, the main regulator of adenosine levels, results in alterations in methylation-dependent gene expression regulation mechanisms that are essential for fetal programming [22].

Substance P (SP) is a member of the tachykinin (TK) peptides family and mediates activation of the neurokinin 1 receptor (NK1R), thus modifying multiple pathophysiologic processes in the organism [23]. SP is a neurotransmitter secreted by nerve endings, various non-neuronal cells, monocytes, and macrophages [24]. SP can be found in the endothelium and myocytes of fetal blood vessels, decidua, and trophoblast [24], as well as the umbilical cord, fetal membranes, and placenta [25]. Those synthesized in the Hofbauer cells induce cell proliferation and neo-angiogenesis and thus the blood flow [24]. Hofbauer cells, which are placental macrophages in the chorionic villous stroma, close to fetal capillaries, regulate placental vasculogenesis and fusion of cytotrophoblast and syncytia. Moreover, Hofbauer cells stimulate trophoblast growth and differentiation [24]. Through NK1R activation, SP acts regulatorily in an autocrine, paracrine, and endocrine manner [24]. In general, SP stimulates secretion in the gastrointestinal and pulmonary systems, leads to smooth muscle contraction, and has special effects on the central nervous system (CNS) [26]. SP acts as a neuronal sensory transmitter in pain and central responses to stress and anxiety. It is known that SP is an important factor in obesity development, and there are several reports on the potential role of SP in insulin signaling dysregulation [26,27]. Fu et al. investigated associations between SP, interleukin 6 (IL-6), and c-reactive protein (CRP) with T2DM and found higher SP, IL-6, and CRP concentrations, higher values of homeostatic model assessment-insulin resistance (HOMA-IR) in obese patients with T2DM and a positive correlation between body mass index (BMI), logHOMA-IR, and SP [27]. However, the overall effect of SP on diabetes may be ambivalent, with studies reporting both diabetogenic and anti-diabetogenic effects [23]. SP is considered to be a crucial neurotransmitter in the pancreas that additionally influences pancreatic secretion [23]. Murine models showed that individuals with NK1R deficiency when fed with a high-fat diet had lower weight gain and reduced levels of circulating leptin and insulin in their blood [28].

**Figure 1:**
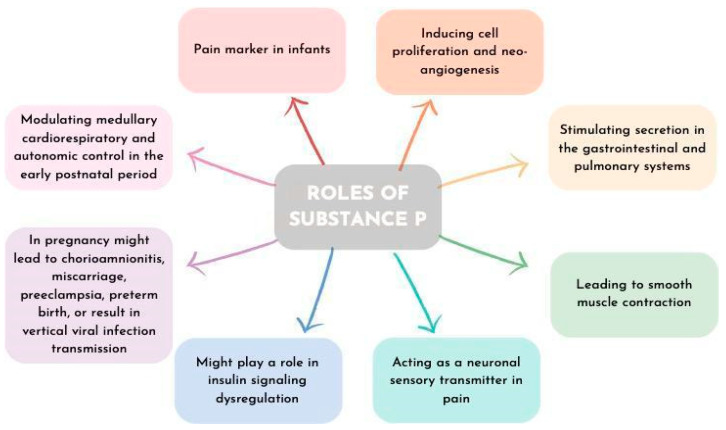
Roles of substance P.

SP/NK1R impairment in pregnancy could lead to several pathologies including chorioamnionitis, spontaneous abortion, miscarriage, preeclampsia, preterm birth, or result in vertical viral infection transmission (i.e., human immunodeficiency virus (HIV)) [24,25,26]. In infants, SP is a pain marker, and its levels increase when invasive procedures are performed. Crucially, SP along with NK1R plays a key role in modulating medullary cardiorespiratory and autonomic control in the early postnatal period [29]. Lately, there have been reports of the potential use of SP properties in the treatment of chronic wounds [30]. The roles of SP are presented in Figure 1.

In essence, fetal programming refers to the process whereby factors in the uterine environment influence fetal development, with consequences observable even in adulthood. One such factor is SP, which, apart from its numerous physiological roles, is also linked to dysregulation of insulin metabolism, increased body mass, and pregnancy pathologies. Understanding the role of this peptide in fetal programming could be crucial, offering new insights into short- and long-term patient care.

Recognizing the significance of obesity and the still limited knowledge and understanding of the fetal programming background, we aimed to explore the role of SP in these processes. In this article, we present the results of our study on the effect of SP levels in patients with GDM and EGWG on the birth parameters of their offspring.

## 2. Results

**Figure 2:**
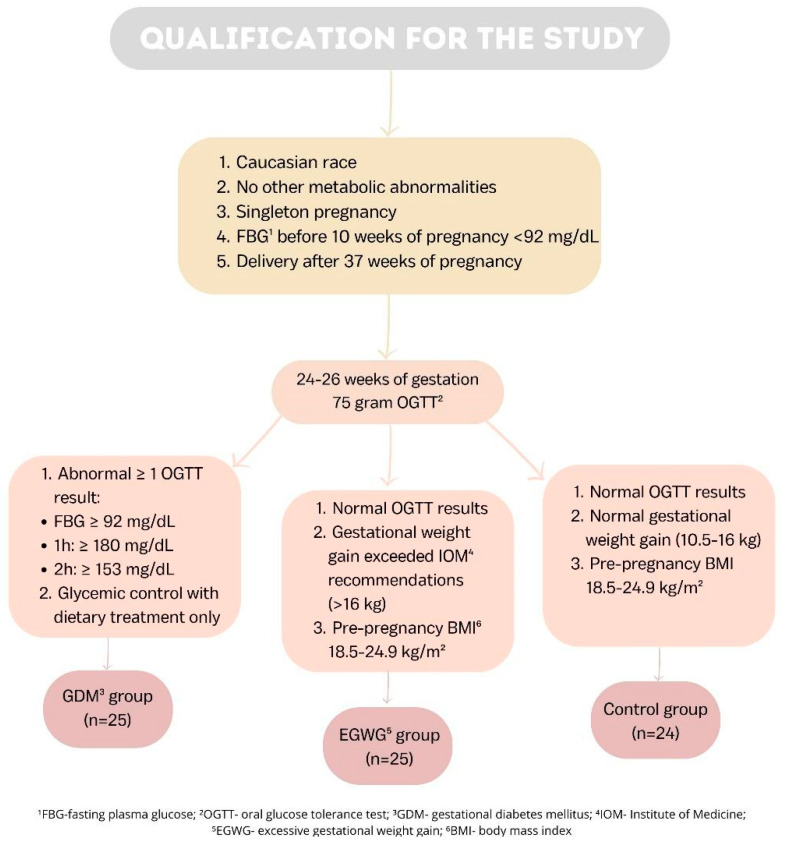
Qualification for the study.

Three groups of seventy-four patients in total participated in our study. Twenty-five women with at least one abnormal OGTT measurement between 24 and 26 weeks of pregnancy were included in the GDM group. Those women had the highest pre-pregnancy BMI of all the groups, and they received diet-only treatment. Twenty-five women whose pre-pregnancy BMI was normal were recruited into the EGWG group; nevertheless, based on the Institute of Medicine (IOM) criteria [31], their gestational weight increase was excessive. The patients in this group did not fit the requirements for a GDM diagnosis. The control group consisted of 24 women who presented normal pre-pregnancy BMI and GWG and were not diagnosed with GDM. The recruitment criteria for our study are presented in Figure 2.

A comparison of variables for all three groups included in our study is summarized in Table 1.

Comparing SP concentrations in serum on delivery day and umbilical cord blood, there were statistically significant differences (*p* > 0.05) between mothers with GDM, patients with EGWG, and controls (Table 2).

The first parameter considered was the correlations of SP determinations in serum on delivery day and umbilical cord blood. A statistically significant relationship was found (*p* < 0.05) when we considered all groups simultaneously (GDM, EGWG, and control group) but also the GDM group independently. In both cases, the correlation is positive, so the higher the serum SP concentration, the higher the concentration in umbilical cord blood. And vice versa, the higher the concentration in umbilical cord blood, the higher the concentration in serum (Figure 2). However, when considering the EGWG group and the control group separately, the relationship was statistically insignificant (*p* > 0.05).

The second considered parameter was the correlation between the baby’s birth weight and the patient’s weight gain during pregnancy. Our statistical analysis showed a significant correlation *p* < 0.05) in the EGWG group. The correlation is negative, so the higher the patient’s gestational weight gain, the lower the baby’s birth weight. And vice versa, the higher the baby’s birth weight, the lower the patient’s gestational weight gain. In all groups, GDM group and control group, there was no statistically significant correlation (*p* > 0.05).

**Figure 3:**
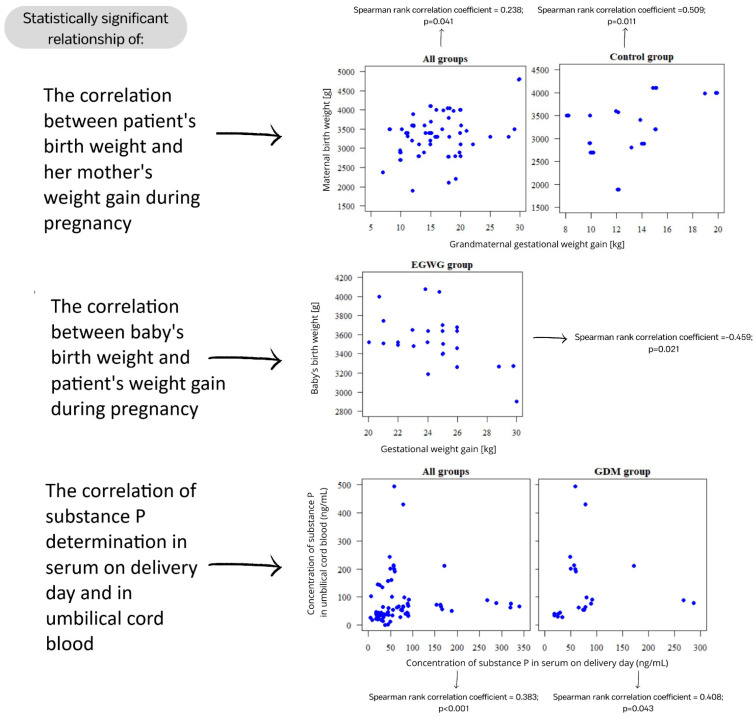
Statistically significant relationship of correlations in our study.

The last parameter considered was the correlation between the patient’s birth weight and her mother’s weight gain during pregnancy. A statistically significant correlation was found (*p* < 0.05) when we considered all groups simultaneously (GDM, EGWG, and control group) and also the control group independently. The positive correlation means that the higher the grandmaternal gestational weight gain, the higher the maternal birth weight. There was no statistically significant relationship (*p* > 0.05) between the GDM group and the EGWG group. Figure 3 illustrates the correlations depicted by the results.

**Figure 4:**
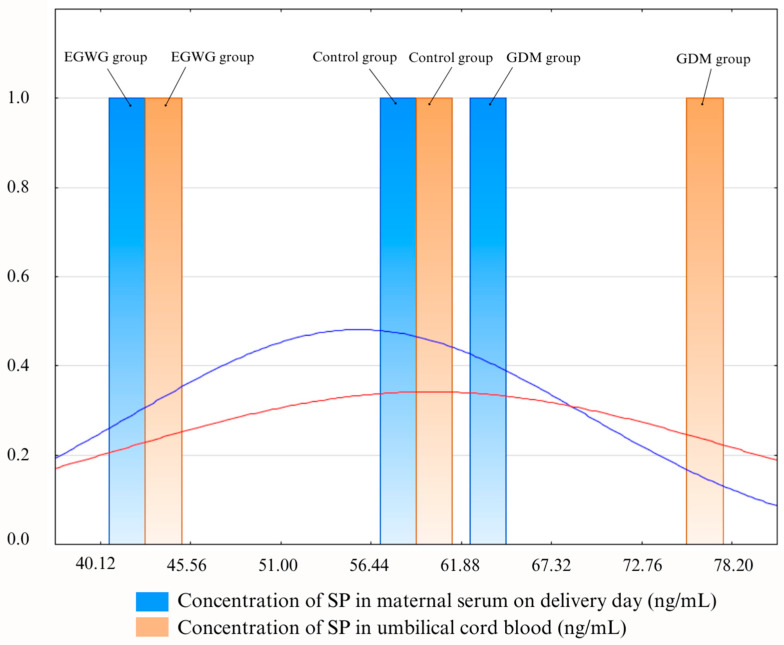
Histogram illustrating the results. GDM group—group of patients with gestational diabetes mellitus; EGWG group—group of patients with excessive gestational weight gain; control group—control group of patients; SP—substance P.

The difference between the values obtained in the three groups is illustrated as a histogram in Figure 4.

## 3. Discussion

In pregnancies complicated by maternal obesity, EGWG or GDM, the placenta is exposed to environmental changes such as increased inflammation and oxidative stress, dyslipidemia, and altered hormone levels. These changes can affect placental development and function and lead to abnormal fetal growth and development, as well as metabolic and cardiovascular disorders in the offspring. Current literature emphasizes the importance of transgenerational epigenetic programming, especially in the context of lifestyle diseases such as diabetes and obesity [32,33,34,35,36]. For this reason, we determined the medical history of our patients, including the frequency of diabetes and obesity in their family, but also the gestational weight gain achieved by their mothers. The latter parameter correlated positively with the birth weight of our patients.

In our research, to our knowledge, we were the first to find increased levels of SP in cord blood in women with GDM compared to the EGWG group and the control group.

GDM is one of the most common complications of pregnancy and is increasing in global prevalence. The condition is associated with numerous health issues affecting pregnant women, such as an increased incidence of gestational hypertension, preeclampsia, and preterm labor. Furthermore, a large percentage of such pregnancies require delivery by cesarean section. Persistently elevated maternal blood glucose also affects the developing fetus, causing excessive production of fetal insulin, resulting in a higher incidence of macrosomia and birth trauma [37,38]. Many studies have found an increased risk of macrosomia also in children of mothers with EGWG [39,40,41,42]. In our study, we found no differences in the anthropometric examination of the newborns in the three study groups: children born to healthy mothers, those with EGWG, and GDM. The only statistically significant result is the negative correlation found in the EGWG group between maternal gestational weight gain and the baby’s birth weight.

Our study aimed to evaluate the effect of SP concentrations on the birth parameters of children in a group of women with GDM and EGWG. To the best of our knowledge, there are no studies investigating the association of SP with GDM and EGWG. In our research, we determined the correlation of SP in maternal blood and umbilical cord blood. We did not observe significant differences in maternal blood SP concentrations, in all study groups. All samples were collected from women in all three groups on the day of delivery.

**Figure 5:**
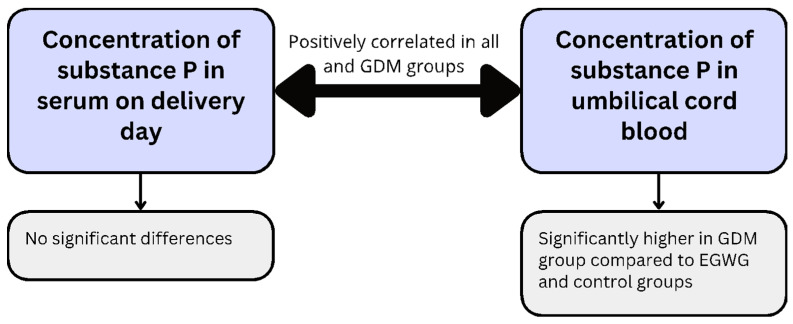
Graphical representation of the results.

The concentration of SP in cord blood was significantly higher in the GDM group compared to the EGWG group and the control group. Also, we observed a positive correlation between SP concentration in cord blood and maternal blood in the GDM group (Figure 5). As is well known, the pathogenesis of GDM is not fully understood. In obesity and T2DM, abnormal insulin signaling is an important factor mediating the increase in insulin resistance. Clinical studies have shown that a chronic increase in SP may represent a strong risk for the development of insulin resistance, which leads not only to T2DM but also to GDM.

SP has been found in several cellular systems that also have neurokinin-1 receptors. It is present in the central and peripheral nervous system and the immune system. SP has multiple functions, acting as a neuronal sensory relay associated with pain and central responses to stress and anxiety. In addition, SP plays a role in enhancing the inflammatory response in the immune system. The interaction of SP with neurokinin 1 receptor (NK-1R) leads to the activation of NF-κB and increased production of pro-inflammatory cytokines, including interleukin 1 (IL-1), IL-6, TNF-α, macrophage inflammatory protein 1β (MIP-1β), and IFN-γ. It is involved in signal transduction in the inflammatory pathway in human preadipocytes. This process is similar to the chronic subacute low-grade inflammatory response found in the adipose tissue of obese individuals [27,43]. Studies have shown that activation of inflammation has a significant impact on the development of insulin resistance and T2DM. Subclinical inflammation increases the risk of T2DM through insulin resistance, which is closely associated with obesity [26]. Excessive weight gain during pregnancy leads to obesity, which is a factor in the development of insulin resistance. SP may be involved in the pathophysiology of GDM by a similar mechanism as in T2DM [44]. It has been suggested that SP may be involved in the Coronavirus disease 2019 (COVID-19) complications. Mehboob named SP as a possible factor responsible for the initiation of the cytokine storm after COVID-19 infection. In addition, SP probably also caused micro-vessel permeability and the development of inflammation, resulting in increased severity of the disease course [45].

SP is expressed in the human placenta. It is present in syncytiotrophoblast and fetal membranes, endothelial cells, and myocytes of fetal blood vessels. Knowing the exact role of SP in the human placenta requires conducting further research. It may play a role in the regulation of local blood flow, as well as in placental formation due to its ability to induce cell proliferation and neoangiogenesis. In addition, dysregulation of the SP/NK-1 receptor system may be involved in pregnancy pathology, such as miscarriage, preeclampsia, preterm labor, and chorioamnionitis [24].

It has been well known that there is a relationship between infants of women with diabetes during pregnancy and the occurrence of respiratory distress syndrome (RDS) [46,47]. Atar et al. [48] noted that fetal hyperglycemia and hyperinsulinemia secondary to maternal diabetes may disrupt lipid and protein surfactant synthesis, leading to surfactant deficiency and subsequent respiratory distress in newborns. The exact pathomechanism of this phenomenon remains unknown. However, it seems that SP may be a key molecule in understanding why newborns, especially premature ones, of diabetic mothers are at risk for RDS.

In the study by Bryndina et al. [49], with a single injection of SP, the researchers achieved a reduction in surfactant disruption. Only with multiple injections of SP did they obtain the opposite effect. This may indicate that only long-term elevated concentrations of SP affect surfactant synthesis and function. Undoubtedly, the long-term effects of various molecules, including SP, can be expected in the intrauterine life of fetuses of diabetic mothers.

Rice et al. [50] investigated SP function in the respiratory system by isolating type II epithelial cells from the lungs of adult rats. They found that the NH2-terminal basic groups of SP inhibit surfactant secretion from isolated type II cells. De Angelis et al. [51] investigated by electrospray ionization mass spectrometry that SP hydrolyses in aqueous surfactant according to dichotomous kinetics. It is initially fast and then slows down as the reaction proceeds. An interesting point was raised by Bright et al. [29], who found an extensive distribution of SP and NK1R in medullary nuclei closely associated with cardiorespiratory function and autonomic control. Interestingly, significantly higher NK1R-binding was observed in preterm and male infants. Sex differences in brainstem neurotransmitter expression may be directly related to disorders in which the death rate in males is much higher than in females, particularly deaths related to respiratory conditions.

In another study, Bright et al. investigated the relationship between the SP/NK1R system in the medulla and the occurrence of sudden infant death syndrome (SIDS). As SP/NK1R is involved in the regulation of respiratory rhythm generation and the integration of cardiovascular control, abnormalities in SP neurotransmission may be the cause of autonomic dysfunction during sleep and contribute to SIDS deaths. SIDS cases presented an altered abnormal developmental profile of the SP/NK1R system in the core, compared to controls [52].

In our study, the postnatal Apgar score of the newborns of mothers with GDM did not differ from those of the other two groups, although the study was carried out only in women giving birth once (at term pregnancy). In addition, the patients recruited to the GDM group in our study were treated only with a diabetic diet and were metabolically balanced.

Our pilot study—conducted in three small study groups—has several limitations due to this fact. For this reason, we chose not to compare the results of cord blood SP concentrations in each group in terms of the sex of the children. Bright et al. [53] found higher SP concentrations in the serum of male offspring. Thus, it would certainly be of great interest to determine the differences according to the sex of the children of mothers with GDM in particular.

Undoubtedly, our study highlights the need for further research in different research groups of this extremely interesting molecule, which came back into the spotlight since the COVID-19 pandemic. Given the association of SP with surfactant production, regulation of respiratory rhythm, and participation in the cytokine storm, we hope that our surprising result of higher SP concentrations in cord blood in full-term newborns without any respiratory disorders, born to GDM mothers, will inspire more than just us.

## 4. Materials and Methods

Seventy-four Caucasian females in a singleton term pregnancy (after 37 weeks of gestation) who gave birth at the Medical University of Lublin’s Chair and Department of Obstetrics and Perinatology were included in this study. The patients were divided into three groups: twenty-five women with GDM formed the first group, twenty-five women with EGWG created the second group, and twenty-four women with normal singleton term pregnancy, free of additional metabolic abnormalities and risk factors, made up the third group.

Because GDM frequently coexists with other disorders, such as gestational hypertension, the qualification process has been rendered substantially more rigorous because all patients included in our study must not have been diagnosed with other chronic or gestational diseases.

First, each pregnant woman was tested by a fasting plasma glucose test before 10 weeks of pregnancy, usually at the first examination during pregnancy. The 75 g oral glucose tolerance test (OGTT) was conducted between 24 and 26 weeks of pregnancy if the test result was less than 92 mg/dL. This test, which is advised for the identification of GDM in compliance with the Minister of Health’s Regulation [53], which has been in effect in Poland since 1 January 2019, was utilized in selecting our GDM group. The first examination during pregnancy should request one if the patient has a history of GDM, if there are risk factors for GDM such as obesity or macrosomia, or if the result of a fasting plasma glucose test performed before 10 weeks of pregnancy is greater than 92 mg/dL. The following ranges of measurement findings permitted us to diagnose GDM: fasting blood glucose ≥ 92 mg/dL, at 60 min ≥ 180 mg/dL, and at 120 min ≥ 153 mg/dL. According to the 2009 guidelines of the IOM [31], a patient who has not been diagnosed with GDM but has gained over 16 kg during pregnancy and has a normal BMI before becoming pregnant is eligible for the EGWG category. Those women in the control group had a gestational weight gain of 11.5–16 kg and were not diagnosed with GDM.

Each patient enrolled in our study provided written consent after receiving information about the study’s protocol. The study protocol received approval from the Bioethics Committee of the Medical University of Lublin (KE-0254/61/2020, approved on 26 March 2020).

We measured many parameters in our study including weight before pregnancy, weight before delivery, glycemia before 10 weeks of pregnancy, gestational weight gain, and parameters of the newborn: birth weight, birth length, pulsatility index, head circumference, chest circumference, and Apgar’s score in the first and fifth minute of the newborn’s life.

SP concentrations were determined in maternal serum and umbilical cord blood samples taken on the delivery day using an enzyme-linked immunosorbent assay (Sandwich ELISA) and kits available on the market (R&D Systems, Inc., Minneapolis, MN, USA; Substance P Parameter Assay Kit; catalog number KGE007; detection range 39.0–2500 pg/mL, sensitivity 43.8 pg/mL).

In our study, we used the Chi-squared test (with Yates correction for 2 × 2 tables) or the Fisher exact test (in case of low expected values) for comparisons of qualitative variables between groups. The Mann–Whitney test was used for comparisons of quantitative variables between two groups. Spearman’s correlation coefficient was used to assess the correlation between two quantitative variables. The significance level was set to 0.05. All the analyses were conducted in R software, version 4.3.1.

## 5. Conclusions

Our research revealed significantly higher SP concentration in umbilical cord blood in the GDM group compared to EGWG and control groups. We believe that even though neonatal anthropometry showed no significant differences in any of the studied groups, SP might be associated with fetal development and its elevated levels in umbilical cord blood might reflect maternal metabolic impairment occurring during a pregnancy complicated by GDM. SP concentration in maternal serum on delivery day showed no significant differences between the studied groups, which suggests that testing its levels is not useful in assessing metabolic disorders in pregnant women.

Further studies are needed to evaluate the utility of SP as a marker of metabolic disorders in fetal development complicated by maternal GDM. In our opinion, a very interesting direction for future research is the relationship between the concentration of SP in newborns of diabetic mothers and the occurrence of respiratory distress syndrome as a complication of impaired surfactant synthesis. We hope that our pilot study will provide direction for future research on larger populations.

## Figures and Tables

**Table 1:** Comparison of characteristics of the study subjects.

**a. Maternal**					
**Variables**		**GDM Group** **n = 25** **A**	**EGWG** **n = 25** **B**	**Control Group** **n = 24** **C**	***p***
Age (years)	Median (quartile)	30 (27–32)	28 (25–30)	28 (25–29.25)	*p* = 0.071
Weight before pregnancy (kg)	Median (quartile)	73.9 (66–78)	62 (59–66.5)	60 (56.75–65.78)	*p* < 0.001 * A>B,C
Weight before delivery (kg)	Median (quartile)	83.3 (75–95)	86 (83–92)	72.35 (70–78.25)	*p* < 0.001 * B,A>C
Glycemia before 10 weeks of pregnancy (mg/dL)	Median (quartile)	89 (86–90)	80 (78–84)	84.5 (79–89.25)	*p* < 0.001 * A>C>B
GWG (kg)	Median (quartile)	11 (8.5–14)	24.80 (23–26)	13 (8–15)	*p* < 0.001 * B>C,A
Maternal weight (g)	Median (quartile)	3400 (3100–3600)	3300 (3100–3500)	3300 (2867.5–3695)	*p* = 0.719
Maternal GWG (kg)	Median (quartile)	14 (12–17)	18 (16–20)	12.5 (10–15)	*p* < 0.001 * B>A,C
**b. Neonatal**					
**Variables**		**GDM Group** **n = 25** **A**	**EGWG** **n = 25** **B**	**Control Group** **n = 24** **C**	***p***
Birth weight (g)	Median (quartile)	3270 (3150–3700)	3520 (3400–3650)	3540 (3230–3905)	*p* = 0.207
Birth length (cm)	Median (quartile)	54 (53–56)	55 (54–56)	55 (55–56)	*p* = 0.058
PI	Median (quartile)	2.08 (2–2.3)	2.1 (2–2.33)	2.10 (1.94–2.21)	*p* = 0.84
Head circumference (cm)	Median (quartile)	34 (34–35)	34 (34–35)	34.5 (34–35)	*p* = 0.441
Chest circumference (cm)	Median (quartile)	35 (33–35)	34 (33–35)	34 (33.75–35)	*p* = 0.942
Apgar 1’	Median (quartile)	10 (9–10)	10 (10)	10 (10)	*p* = 0.764
Apgar 5’	Median (quartile)	10 (10)	10 (10)	10 (10)	*p* = 0.223

*p*—qualitative variables: Kruskal–Wallis test + post-hoc analysis (Dunn test); *—difference statistically significant (*p* < 0.05). A—group of patients with gestational diabetes mellitus; B—group of patients with excessive gestational weight gain; C—control group of patients; GDM—gestational diabetes mellitus; EGWG—excessive gestational weight gain; PI—pulsatility index; GWG—gestational weight gain.

**Table 2:** Comparison of SP concentration.

Parameter		GDM Group n = 25 A	EGWG n = 25 B	Control Group n = 24 C	*p*
Concentration of SP in serum on delivery day (ng/mL)	Median (quartile)	64.94 (48.2–80.4)	40.12 (24.78–56.9)	61.85 (36.53–105.89)	*p* = 0.057
Concentration of SP in umbilical cord blood (ng/mL)	Median (quartile)	78.2 (53.5–199.45)	40.2 (34.2–56.51)	61.19 (34.77–69.59)	*p* = 0.006 * A>C,B

*p*—Kruskal–Wallis test + post-hoc analysis (Dunn test); *—difference statistically significant (*p* < 0.05). A—group of patients with gestational diabetes mellitus; B—group of patients with excessive gestational weight gain; C—control group of patients; GDM—gestational diabetes mellitus; EGWG—excessive gestational weight gain; SP—substance P.

## Data Availability

The data presented in this study are available on request from the corresponding author.
